# Determine the Compressive Strength of Calcium Silicate Bricks by Combined Nondestructive Method

**DOI:** 10.1155/2014/829794

**Published:** 2014-09-03

**Authors:** Jiri Brozovsky

**Affiliations:** Faculty of Civil Engineering, Brno University of Technology, Institute of Technology of Building Materials and Components, Veveri 331/95, 60200 Brno, Czech Republic

## Abstract

The paper deals with the application of combined nondestructive method for assessment of compressive strength of calcium silicate bricks. In this case, it is a combination of the rebound hammer method and ultrasonic pulse method. Calibration relationships for determining compressive strength of calcium silicate bricks obtained from nondestructive parameter testing for the combined method as well as for the L-type Schmidt rebound hammer and ultrasonic pulse method are quoted here. Calibration relationships are known for their close correlation and are applicable in practice. The highest correlation between parameters from nondestructive measurement and predicted compressive strength is obtained using the SonReb combined nondestructive method. Combined nondestructive SonReb method was proved applicable for determination of compressive strength of calcium silicate bricks at checking tests in a production plant and for evaluation of bricks built in existing masonry structures.

## 1. Introduction

Nondestructive testing methods are used for determining the strength of building materials and products. These usually include the rebound hammer method and the ultrasonic pulse method. Strength is determined from the calibration relation between a parameter obtained from nondestructive tests and compressive strength. Compressive strength can be assessed either separately for each used method and/or on the basis of several parameters from nondestructive tests (combined nondestructive methods).

Nondestructive testing methods are used to the largest extent for assessment of concrete quality built in a structure; testing and assessment procedures are codified in technical standards, for example, ISO 1920-7 [1], ASTM C805 [2], ASTM 597 [3], CSN EN 12504-2 [4], and CSN EN 12504-4 [5].

Knowledge about nondestructive testing of concrete with rebound hammer and ultrasonic pulse method is stated in various technical literatures, like [6–9]. Rebound hammer and ultrasonic pulse method are also used for testing of other construction materials. Knowledge about testing cement and concrete paving blocks with nondestructive methods (ultrasonic pulse method: cement and concrete paving blocks) is stated, for example, in [10, 11]; testing concrete paving blocks with rebound hammer is stated in [10]. Problems of determination of compressive strength of bricks (clay brick, calcium silicate brick) are described, for example, in Aliabdo and Elmoaty [12], Brozovsky [13], and McCann and Forde [8].

Measurement with rebound hammer and ultrasonic pulse method is influenced by various factors, for example, components and composition of tested material, moisture content, temperature of the material during testing, state of the surface of tested area, inner defects of the material, or position of sounders for ultrasonic pulse measurement.

Most of knowledge about influence of various factors on results of measurement with nondestructive methods for concrete is stated in [6, 7, 14]. The author examined selected factors with influence on measurements with ultrasonic pulse method and rebound hammer for calcium silicate bricks and clay bricks; the knowledge is given in publications [15–17]. For the method of rebound hammer, influence of temperature and moisture content of bricks was observed [16] as well as the influence of internal tension of the specimen on the rebound value [17], for the ultrasonic pulse method and the influence of moisture content and mode of sounding (direct, semidirect) [15].

The combined nondestructive methods are based on two or more nondestructive methods. More parameters obtained from these methods define the monitored property of a material or product more accurately. For example, the strength characteristics of concrete are influenced by various factors, such as concrete components, precision of batching, production technology, the manner of processing and climatic conditions during concrete pouring, and how the hardened concrete is treated.

For tests using the combined nondestructive method, if there is a certain degree of correlation dependence between two variables, it can be assumed that multiple correlation dependence between one and two variables will show a higher degree of correlation.

Application of the combined nondestructive method is based on tests using the rebound hammer method and ultrasonic pulse method, which is also called the SonReb method and is developed mostly for determining compressive strength of concrete. Testing methodology is specified in the NDT 4 RILEM directive [18]; relationships for determining compressive strength from ultrasonic pulse velocity and the rebound number are quoted in a series of professional publications, for example, [19–23].

This paper provides information on testing of calcium silicate bricks (CSB) using the combined method, specifically the combination of ultrasonic pulse method and rebound hammer method (SonReb).

## 2. Materials and Methods

### 2.1. Basic Characteristics of Calcium Silicate Bricks

The basic raw materials for the production of calcium silicate units are airy lime and silica sand. A very important element of the production of sand-lime brick is called autoclaving. During this process, dried bricks are loaded into an autoclave where they are exposed to synergistic effect of elevated temperature (the temperature range is 170°C to about 195°C) and adequate pressure saturated steam (pressure approximately 16 bars). Course of the heating, dwell times and steam pressure is selected according to parameters of raw materials. Effect of boundary conditions defined in this way creates an environment for the emergence of the so-called hydrothermal reaction. Under these specific conditions silicon dioxide reacts significantly with the calcium oxide. The products of hydrothermal reactions are mainly the calcium hydrosilicate phases. These phases could be crystalline or gel (de facto amorphous phase). The most significant product of autoclaving is tobermorite. Other holders of the mechanical properties of autoclaved matrices are xonotlite (summarily C_5_S_5_H), respectively, afwillite, and so forth.

Measurements were taken on two sets of calcium silicate bricks from various times of production; test results with nondestructive methods are influenced by technological factors (pressing force, density of the structure, and defects in the inner structure).

Calcium silicate bricks of the format 290 × 140 × 65 mm were used for experiments; they featured the following basic parameters:

- compressive strength *f*_*c*_ ∈ {16.5; 61.5 MPa},
- density of dried bricks *D* ∈ {1661; 1874 kg/m^3^},
- number of calcium silicate bricks in a set—140.

### 2.2. Experimental Method

*Test Equipment*. The L-type Schmidt rebound hammer (initial impact energy of 0.735 Nm); TICO ultrasonic instrument, accuracy of 0.1 *μ*s, and natural frequency of transducers 82 kHz (the given frequency of transducers was selected so that the wave length ratio *λ* to the least sample size in the direction of direct sound transmission *d*_min⁡_ was less than 1 for reasons of limiting any lowering of ultrasonic pulse velocity. Ultrasonic pulse velocity in tested samples ranged between 1.6 and 3.1 km/s; the ratio *λ*/*d*_min⁡_ corresponds to these values, which equals 0.30, respectively, 0.58, that is, the given condition was complied with).

*Condition of Samples during Testing*. They are dried to a constant weight. 

*Rebound Hammer Method (RM)*. A test sample was placed in the compression test machine and loaded by a force corresponding to 10% of the final compressive strength. 24 measurements of rebound numbers *R*_*i*_ were taken on each brick. The average *R* was calculated from respective rebound numbers *R*_*i*_ obtained from the test sample, and such numbers that differed from the average by more than 14% were excluded (the given limit for outlaying values is based on the analysis quoted in [10]). Minimum count of valid numbers for the sample to be classified into the assessed set was 20. 

**Figure 1:**
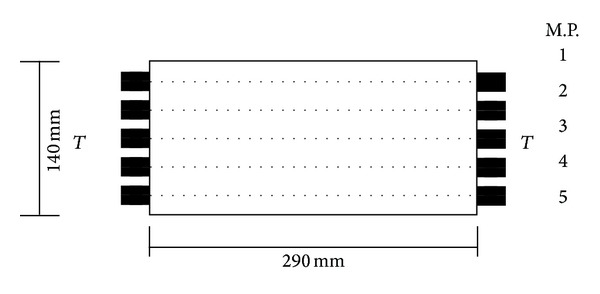
Scheme of direct sound transmission CSB.

*Ultrasonic Pulse Method (UPM)*. Measurements were taken by direct sound transmission along the length of a calcium silicate brick in 5 measuring points (M.P.); see Figure 1.

Ultrasonic pulse velocity was calculated from

(1) $$\begin{array}{c} V=\frac{L}{T}, \end{array}$$

where *V* is ultrasonic pulse velocity (km/s), *L* is length of measuring base (mm), and *T* is transit time (*μ*s).

*Compressive Strength.* It was determined on bricks after nondestructive methods were measured according to CSN EN 772-1 [24].

## 3. Results and Discussion

**Figure 2:**
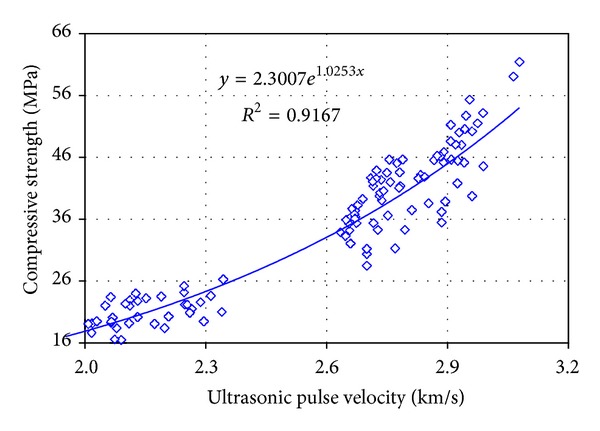
Results of measurements using the ultrasonic pulse method; relationship between ultrasonic pulse velocity and compressive strength of calcium silicate bricks.

**Figure 3:**
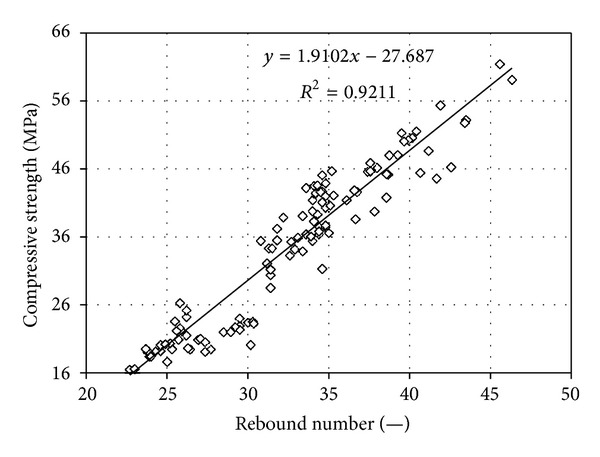
Results of measurements using the L-type Schmidt rebound hammer; relationship between the rebound number and compressive strength of calcium silicate bricks.

A summary of test results is given in Table 1 and, for respective methods, they are indicted in Figures 2 and 3.

### 3.1. Relationships for Prediction of Compressive Strength from NDT

Based on test results, using the least square method, the relationships (2)–(4) for determining compressive strength were obtained from the nondestructive test parameters:

 
*rebound hammer Schmidt, type L, is*
  (2) $$\begin{array}{c} f_{c,L}=1.9102R-27.687\;r=0.96; \end{array}$$
 
*ultrasonic pulse method is*
  (3) $$\begin{array}{c} f_{c,U}=2.3007e^{1.0253V}\;r=0.96; \end{array}$$
 
*combined method (SonReb) is*
  (4) $$\begin{array}{c} f_{c,SR}=0.255082V^{1.246413}R^{1.0559}\;r=0.97. \end{array}$$

To assess the applicability of processed relationships, the residual standard deviation was determined, which expresses the difference between the compressive strength value determined destructively and the compressive strength value calculated from the respective relation. The residual standard deviation *S* for *n* measured points of the calibration relationship is calculated according to

(5) $$\begin{array}{c} S=\sqrt{\frac{\sum_{i=1}^{n}{\left(D_{i}-D_{m}\right)}^{2}}{n-k}}, \end{array}$$

where *D*_*i*_ is calculated according to (6) and *D*_*m*_ according to (7) as

(6) $$\begin{array}{c} D_{i}=\left|\frac{f_{ci}-f_{cdi}}{f_{cdi}}\right|, \end{array}$$

(7) $$\begin{array}{c} D_{m}=\frac{\sum_{i=1}^{n}D_{i}}{n}, \end{array}$$

where *f*_*ci*_ is compressive strength for the *i*th measured point of calibration relationship determined using the destructive test; *f*_*cdi*_ is compressive strength calculated from the nondestructive measurement parameter obtained from the calibration relationship for the *i*th point; *n* is number of measured points of calibration relationship; and *k* is number of parameters of a free calibration relation function.

Those relationships are regarded as practically applicable in which *S* ≤ 0.12 and which have no local minimum or maximum inside their range.

Residual standard deviations of processed relationships (2)–(4) for the prediction of compressive strength of calcium silicate bricks obtained from the nondestructive test parameter are given in Table 2.

### 3.2. Discussion of the Results

The processed relations for the prediction of compressive strength obtained from the nondestructive test parameter are known for close correlation between variables, irrespective of whether or not the compressive strength was calculated from the relation with one or two parameters taken from nondestructive tests. Correlation coefficients are within 0.96 to 0.97 and residual standard deviations are within 0.043 to 0.066. The closest relationship between variables is found in the relation processed from the results of measurements using the combined method (combination of ultrasonic pulse and rebound method).

**Figure 4:**
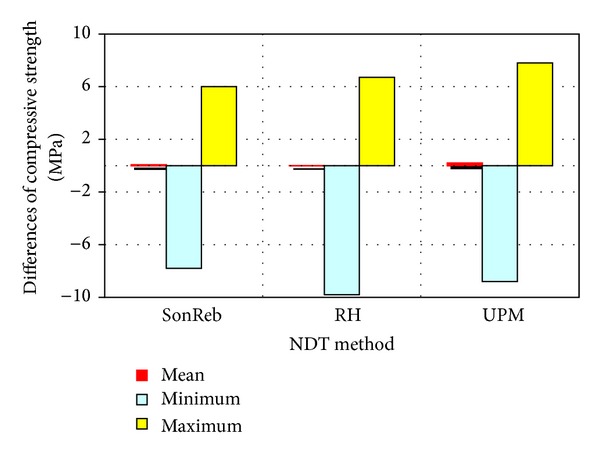
Comparing the differences between the actual compressive strength and compressive strength obtained from nondestructive tests.

Comparison of differences between the actual compressive strength (compression test machine) and the strengths calculated from calibration relations processed for respective methods is indicated in Figure 4.

For the SonReb combined method, the differences between the actual compressive strength and compressive strength obtained from nondestructive tests lie in the interval −7.8 to 6.0 MPa; for the ultrasonic pulse method, they are in the interval −8.8 to 7.8 MPa and, for the rebound hammer method, in the interval −9.8 to 6.7 MPa.

Differences in predicted compressive strengths of calcium silicate bricks determined in accordance with relations processed for respective methods are lower than in concretes, which can be explained by a lower parameter variability of components of calcium silicate raw material, by substantially invariable parameters of production technology, by a higher homogeneity of calcium silicate material, and by definitely defined moisture condition of samples prior to testing.

## 4. Conclusion

Based on the analysis obtained from the information and processed relationships needed for the prediction of compressive strength of calcium silicate bricks obtained from tests using the ultrasonic pulse method and the rebound hammer method, it is possible to state that the given methods are both separately and in combination applicable for the assessment of strength parameters of calcium silicate bricks.

The highest correlation between parameters from nondestructive measurement and predicted compressive strength was obtained using the SonReb combined nondestructive method. However, the differences in strengths obtained from the combined method compared with the strengths determined from measurements using a sole method (rebound hammer and ultrasonic pulse method) are not so significant as in concrete testing.

The processed calibration relationships are known for a high linkage between variables and are applicable in practice. Combined nondestructive testing SonReb reaches the highest accuracy of the value of expected compressive strength of calcium silicate bricks.

Practical applicability of the SonReb combined nondestructive method was established as a tool used for assessment of compressive strength of calcium silicate bricks, both for checking tests in the production plant and for determination of compressive strengths of bricks built in a structure (walling).

Setting of specifying coefficient is recommended for practical use of the basic calibration relationship for determination of expected strength of calcium silicate bricks from the measurement by means of the SonReb method (4) for particular production plant or for evaluation of calcium silicate bricks built in the structure.

For the purpose of checking tests in the production plant of the CSB, it is recommended to carry on nondestructive measurements and consequent destructive tests of at least 16 bricks with various compressive strengths, which will help set the specifying coefficient.

Specifying coefficient for compressive strength of calcium silicate bricks built in a structure (wall) is set on at least 3 bricks, which were selected on the basis of results of measurement with SonReb method (minimal, average, and maximal compressive strength of the evaluated set). If ultrasonic pulse method uses semidirect transmission method, the minimal number of bricks is 9.

## Figures and Tables

**Table 1:** Test results.

Parameter	Units	Mean	Minimum	Maximum
Compressive strength	[MPa]	32.4	16.5	61.5
Ultrasonic pulse velocity	[km/s]	2.514	1.952	3.078
Rebound number: Schmidt hammer type L	[—]	31.5	22.7	46.3

**Table 2:** Residual standard deviation of relationships for prediction of compressive strength of calcium silicate bricks.

Nondestructive method	Equation	*S *
Rebound hammer Schmidt, type L	(2)	0.066
Ultrasonic pulse method	(3)	0.056
Combined method (SonReb)	(4)	0.043
